# Diagnostic accuracy of artificial intelligence in detecting periapical lesions: A comparative study with cone-beam computed tomography and conventional periapical radiography

**DOI:** 10.4317/jced.64327

**Published:** 2026-09-15

**Authors:** Mays Maher Albochi, Wael Hussein Kamel, Nihal Ezzat Sabet, Marwa Wagih ElBoraey

**Affiliations:** 1 Faculty of Oral and Dental Medicine, Future University in Egypt, Cairo, Egypt; 2 Faculty of Dentistry, Cairo University, Cairo, Egypt

**Keywords:** Artificial intelligence, cone-beam computed tomography, diagnostic accuracy, periapical lesion, radiography.

## Abstract

**Background:**

Periapical lesions, particularly in their early stages, can be challenging to identify clinically and radiographically, as they may be asymptomatic or obscured by anatomical structures on conventional two-dimensional periapical radiography (2D PR). The diagnosis of periapical pathology may also be subjective and influenced by the examiner’s experience, image quality, and interpretation variability. With recent technological advancements, artificial intelligence (AI)-based diagnostic tools have been introduced to support radiographic interpretation and improve the consistency of lesion detection. This study aimed to evaluate the diagnostic accuracy of AI in detecting periapical lesions by comparing its performance with 2D PR and cone-beam computed tomography (CBCT), using CBCT as the reference standard.

**Materials and Methods:**

This diagnostic accuracy study included 177 teeth from adult patients indicated for endodontic treatment. All teeth were examined using conventional 2D PR, CBCT, and AI-based image interpretation. The presence or absence of periapical pathology was recorded for each modality, and CBCT was considered the reference standard. Diagnostic performance was assessed using sensitivity, specificity, positive predictive value, negative predictive value, overall accuracy, receiver operating characteristic (ROC) curve analysis, and agreement testing.

**Results:**

Compared with CBCT, AI showed a sensitivity of 84.16%, specificity of 93.42%, and accuracy of 88.14%. Conventional 2D PR showed lower sensitivity (64.36%) despite high specificity (100%). When periapical radiography was used as reference, AI demonstrated higher sensitivity (89.23%) but lower specificity (71.43%).

**Conclusions:**

CBCT remained the most reliable reference modality for detecting periapical lesions. AI demonstrated promising diagnostic accuracy and may support radiographic interpretation in endodontic practice.

## Introduction

Periapical lesions are among the most common pathological findings encountered in endodontics and are usually associated with pulpal necrosis and microbial infection. Accurate diagnosis is fundamental to successful endodontic treatment, as clinicians are required to interpret clinical findings together with two-dimensional and three-dimensional imaging data to identify pulpal and periapical pathosis and determine the most appropriate treatment approach [1].

Radiographic imaging remains one of the most important diagnostic tools in endodontics. Conventional two-dimensional periapical radiography (2D PR) is widely used because of its accessibility, relatively low cost, and low radiation dose. However, its diagnostic accuracy may be limited by anatomical superimposition, image distortion, and the inability to visualize structures in three dimensions [2].

Cone-beam computed tomography (CBCT) has been introduced as a valuable three-dimensional imaging modality that provides volumetric assessment of dentoalveolar structures [3].

Despite these advantages, interpretation of CBCT and other radiographic images can be challenging and highly dependent on the clinician's experience. The diagnostic process may be affected by observer variability, image quality, lesion size, and the complexity of anatomical structures. Therefore, supportive diagnostic tools that assist clinicians in image interpretation may improve the consistency and efficiency of diagnosis. Computer-assisted systems have gained growing attention for their ability to perform objective tasks such as image segmentation, lesion detection, classification, and quantitative assessment [4].

Artificial intelligence (AI), particularly machine learning and deep learning techniques, has recently emerged as a promising tool in dental and medical imaging. AI-based systems can analyze large datasets, recognize complex image patterns, and support diagnostic decision-making. In endodontics, AI has shown potential in the detection of periapical lesions, assessment of root canal morphology, identification of vertical root fractures, working length determination, and prediction of treatment outcomes [5].

The integration of AI into endodontic imaging may help reduce diagnostic time, minimize human error, and enhance diagnostic accuracy, particularly when lesions are subtle or difficult to detect using conventional methods [6]. 

Recent literature has highlighted the expanding role of artificial intelligence in endodontic imaging and diagnostic workflows. AI-based systems have been investigated for tasks such as radiographic interpretation, automated lesion detection, image analysis, and decision support in clinical endodontics. Ourang *et al*. [7] described the potential integration of AI into different stages of endodontic diagnosis and treatment planning, emphasizing its ability to support clinicians and improve workflow efficiency. Deep learning models have also shown promising performance in detecting periapical diseases on radiographic images and CBCT datasets, as reported by Sadr *et al*., and Kirnbauer *et al*. [8,9]. In addition, AI applications have been evaluated in broader dental radiographic assessments, including panoramic, bitewing, and periapical radiographs, showing potential for improving diagnostic consistency across different imaging modalities. However, despite these encouraging findings, AI performance may vary according to the imaging technique, dataset characteristics, lesion presentation, and validation method used [10,11]. Although AI-assisted detection of periapical lesions has been increasingly investigated, the published evidence remains heterogeneous with respect to imaging modality, study population, reference standard, and software platform. Therefore, further clinical validation is still needed to clarify the diagnostic value of AI in endodontic radiographic interpretation. The present study contributes additional comparative diagnostic data by evaluating a commercially available AI platform against CBCT as the reference standard and comparing its performance with 2D PR in a defined clinical sample. By assessing the diagnostic accuracy and agreement of AI-based interpretation, this study sought to clarify the potential role of AI as an adjunctive tool in endodontic diagnosis.

## Materials and Methods

*Study design and setting*

This diagnostic accuracy study was conducted at the Dental Hospital, Faculty of Oral and Dental Medicine, Future University in Egypt. Data were collected from patients who underwent clinical and radiographic examination between November 2024 and January 2025. Ethical approval was obtained from the Research Ethics Committee of Future University in Egypt, Faculty of Oral and Dental Medicine, under approval number FUE-REC code (52)/11-2024.

The study was conducted in accordance with the Declaration of Helsinki and the Standards for Reporting Diagnostic Accuracy Studies (STARD) guidelines, and it was registered at ClinicalTrials.gov (NCT07319182).

*Sample size calculation*

The sample size was calculated based on the expected diagnostic accuracy of AI in detecting periapical lesions, as reported in a previous diagnostic accuracy study [12]. The calculation was performed using the single-proportion formula n = Z² × P × (1-P) / d², where Z represented the 95% confidence level, P represented the expected accuracy, and d represented the desired precision [12,13]. The minimum required sample size was 51 cases. However, 177 teeth were included to increase the statistical reliability and robustness of the findings.

*Study population*

Eligible participants were adult patients aged 18-50 years with teeth indicated for endodontic treatment and suspected periapical pathology. Inclusion criteria were American Society of Anesthesiologists (ASA) physical status class I or II, restorable teeth, and the ability to provide written informed consent. Patients with poor oral hygiene, pregnant females, or those unable to provide informed consent because of psychological impairment were excluded. 

Teeth were excluded if they were periodontally compromised with grade II or III mobility, had an open apex, or had immature roots.

*Clinical and radiographic examination*

Clinical examination included assessment of pain on percussion and palpation and evaluation of tooth restorability. Teeth presenting with signs and symptoms suggestive of periapical pathology were selected for radiographic evaluation.

All participants underwent preoperative conventional periapical radiographic examination using digital radiography. Subsequently, preoperative CBCT scans were obtained for further evaluation. CBCT images were obtained using a Planmeca ProMax 3D Max machine with a field of view of 8 x 8 cm and a resolution of 0.1 mm and were analyzed using Romexis Viewer software version 3.1.1 (Planmeca, Helsinki, Finland). After establishing the definitive diagnosis, root canal treatment was performed for teeth requiring treatment.

*CBCT image evaluation*

CBCT scans were evaluated in sagittal, coronal, and axial planes to allow three-dimensional assessment of the region of interest. Image datasets were optimized by adjusting brightness, contrast, slice thickness, and interval of cross-sectional images. Image enhancement tools, including interpolation and noise-reduction filters, were applied when required to improve visualization of the periapical region (Figure 1).

Each scan was reviewed dynamically by scrolling through the orthogonal image series. Image reorientation was performed by reformatting the CBCT datasets to align the root axis vertically in both sagittal and coronal planes, ensuring consistent anatomical orientation.  

CBCT images were independently assessed by two experienced endodontists. Before formal evaluation, the examiners were calibrated on a subset of cases not included in the final analysis. Inter-examiner agreement was assessed using Cohen's kappa coefficient. In cases of disagreement, the final interpretation was established by consensus after consultation with an experienced oral and maxillofacial radiologist. 

*AI assessment*

Periapical lesion detection was performed using the Diagnocat AI platform (Diagnocat Inc., San Francisco, CA, USA), a cloud-based deep learning system designed for automated radiological interpretation of dental images, including CBCT datasets [14].

After anonymized CBCT scans were uploaded, the AI software automatically processed the images, segmented the dentoalveolar region, identified individual teeth, and analyzed the periapical bone surrounding each root. The system detected radiographic features suggestive of periapical pathology, including reduced periapical bone density, disruption of lamina dura continuity, and irregular apical contours. 

Healthy periapical bone was characterized by uniform radiodensity and intact lamina dura, whereas suspected lesions were identified by reduced bone density, disrupted lamina dura continuity, and irregular apical contours. For each analyzed tooth, the AI system generated a probability map ranging from 0% to 100%, representing the likelihood of a periapical lesion. In the present study, a confidence threshold of 50% was applied; therefore, values equal to or greater than 50% were classified as positive for periapical pathology (Figure 2) [15].

*Blinding*

Triple blinding was implemented. The researcher interpreted the periapical radiographs and was blinded to the CBCT and AI results. The co-supervisors interpreted the CBCT images while blinded to the periapical radiographic and AI findings. The principal supervisor assessed the AI software-based results, and the statistician was blinded to all radiographic findings, including periapical radiographs, CBCT images, and AI software results.

*Statistical analysis*

Data were collected, tabulated, and statistically analyzed using Microsoft Excel 2016, Statistical Package for Social Sciences (SPSS) version 24.0, and Minitab statistical software version 16. Sensitivity, specificity, positive predictive value, negative predictive value, overall diagnostic accuracy, and their 95% confidence intervals were calculated for the pairwise comparisons between AI, 2D PR, and CBCT. CBCT was considered the reference standard for evaluating the diagnostic performance of AI and 2D PR. Agreement between diagnostic modalities was assessed using Cohen’s kappa coefficient, and differences in lesion detection between methods were evaluated using the chi-square test. Receiver operating characteristic (ROC) curve analysis was performed to evaluate diagnostic performance, and the area under the curve (AUC) was calculated. Inter-examiner agreement for CBCT interpretation was also assessed using Cohen’s kappa coefficient. Statistical significance was set at *P*<0.05.

**Results** 

The diagnostic performance of AI, 2D PR, and CBCT was assessed in 177 teeth. CBCT detected the highest proportion of periapical lesions, followed by AI and conventional 2D PR, indicating lower lesion detection by conventional radiography (Table 1).

The contingency analysis showed that AI had fewer false-negative findings than conventional 2D PR when CBCT was used as the reference standard. Conventional 2D PR missed a considerable number of CBCT-positive lesions, whereas AI showed closer correspondence with CBCT findings (Table 2). When CBCT was used as the reference standard, AI demonstrated higher sensitivity, accuracy, and negative predictive value than 2D PR. Conventional 2D PR showed the highest specificity but lower sensitivity, reflecting a greater tendency to under-detect periapical lesions. When AI was compared with 2D PR, AI detected additional cases not identified by 2D PR, although with lower specificity and positive predictive value (Figure 3, Table 3).

Agreement analysis showed substantial agreement between AI and CBCT, with no statistically significant difference between the two methods. In contrast, 2D PR showed a statistically significant difference from CBCT and only moderate agreement. AI and 2D PR also differed significantly, with moderate agreement between the two methods (Table 4).

ROC analysis confirmed the highest diagnostic performance for AI when CBCT was used as the reference standard, followed by conventional 2D PR against CBCT and AI against 2D PR. All AUC values were statistically significant (Figure 4, Table 4).

Inter-examiner agreement for CBCT interpretation was excellent, with a Cohen’s kappa value of 0.818, indicating high reliability between the two examiners before consensus-based final diagnosis (Table 4).

## Discussion

This study compared the diagnostic performance of AI-based assessment, 2D PR, and CBCT in detecting periapical lesions. The findings showed that AI demonstrated closer agreement with CBCT than 2D PR, whereas 2D PR showed lower sensitivity and missed a considerable number of CBCT-positive lesions. Therefore, the contribution of the present study should be interpreted as additional independent clinical validation within the growing body of evidence on AI-assisted endodontic diagnosis, rather than as a first-time evaluation of AI-based lesion detection.

Although AI-assisted detection of periapical lesions has been previously investigated using both CBCT and 2D PR, the available evidence remains heterogeneous with respect to case selection, image acquisition protocols, reference standards, software platforms, and assessment methods. Diagnostic performance may vary according to lesion characteristics, imaging quality, software architecture, training datasets, and the diagnostic threshold applied. Accordingly, the present study provides further comparative diagnostic data on the performance of a commercially available AI platform in relation to 2D PR and CBCT within a defined clinical sample.

The lower diagnostic performance of 2D PR may be explained by the inherent limitations of two-dimensional imaging, including anatomical superimposition, geometric distortion, and limited representation of three-dimensional periapical bone changes. These findings are consistent with Alsaikhan *et al*. [16], who reported that CBCT is more accurate than conventional periapical radiography in detecting periapical lesions. In contrast, the stronger agreement between AI and CBCT suggests that AI-assisted interpretation may help reduce missed periapical pathology and may serve as an adjunctive diagnostic aid rather than a replacement for clinician judgment or CBCT assessment.

The findings are also consistent with Orhan *et al*. [17], who reported strong agreement between AI systems and CBCT in periapical lesion detection. Similarly, Kazimierczak *et al*. [15] reported high diagnostic accuracy and strong overall performance of AI systems when applied to CBCT imaging. Allihaibi *et al*. [14] also reported high agreement between Diagnocat software and CBCT in detecting periapical lesions, particularly in lesion-free cases. The agreement between the present findings and these studies may be related to the use of CBCT as a reference standard and the application of blinded assessment protocols.

In contrast, Calazans *et al*. [4] reported lower diagnostic accuracy of AI systems than that observed in the present study. This discrepancy may be explained by differences in lesion prevalence, imaging quality, software architecture, training datasets, and the type of images analyzed. Such differences highlight the heterogeneous nature of the current evidence and support the need for further clinical validation of AI systems across different populations, imaging protocols, and diagnostic platforms.

When 2D PR was used as the comparator, AI detected more lesions and showed higher sensitivity, suggesting that AI may be less likely to miss subtle periapical pathology [18]. However, AI also showed moderate specificity and lower positive predictive value, indicating the possibility of false-positive detections. This agrees with Nagareddy *et al*. [19], who reported strong lesion detection by AI systems but noted limitations in precise discrimination. False-positive findings may occur when normal anatomical radiolucencies, periodontal ligament space widening, trabecular patterns, image artifacts, or structures such as the maxillary sinus and mental foramen are misclassified as periapical pathology.

The strengths of the present study include the direct comparison of AI, 2D PR, and CBCT within the same clinical sample, the use of CBCT as the reference standard, and the implementation of blinded assessment. The use of a commercially available AI platform also increases the clinical relevance of the findings, as the results reflect the performance of a system that may be applied in real-world diagnostic workflows.

The present study has several limitations. Only a single AI platform was evaluated, which may limit the generalizability of the findings to other AI systems. In addition, lesion size, lesion location, anatomical complexity, image artifacts, and variations in lesion morphology may have influenced diagnostic performance. Future studies with larger and more diverse datasets, multiple AI platforms, and standardized imaging protocols are recommended to further validate the diagnostic performance of AI in endodontic imaging.

Overall, the present findings suggest that AI-assisted interpretation demonstrated better agreement with CBCT than 2D PR and may serve as a useful adjunctive diagnostic tool for detecting periapical lesions. Nevertheless, AI should not be considered a replacement for clinical judgment or CBCT when three-dimensional assessment is required.

## Conclusions

CBCT demonstrated the highest reliability and diagnostic accuracy for detecting periapical lesions and remained the reference modality for periapical assessment. AI showed promising diagnostic performance and stronger agreement with CBCT than 2D PR. Although AI cannot replace clinician judgment, it may serve as a valuable adjunctive tool for improving diagnostic efficiency and supporting radiographic interpretation in routine endodontic practice.

## Figures and Tables

**Table 1:** Table 1: Distribution of periapical lesion detection across diagnostic methods.

**Diagnostic modality**	**Lesion absent, n (%)**	**Lesion present, n (%)**	**Total, n**
Conventional 2D PR	112 (63.3)	65 (36.7)	177
CBCT	76 (42.9)	101 (57.1)	177
Artificial intelligence	87 (49.2)	90 (50.8)	177

Chi-square test: χ^2^=15.402, df=2, P=0.004. *Statistically significant at P<0.05.

**Table 2:** Table 2: Contingency tables for pairwise comparisons between AI, 2D PR, and CBCT in detecting periapical lesions.

**Comparison**	**Test result**	**Reference/ comparator absent**	**Reference/ comparator present**	**Total**
AI vs CBCT	AI negative	71	16	87
	AI positive	5	85	90
	Total	76	101	177
2D PR vs CBCT	2D PR negative	76	36	112
	2D PR positive	0	65	65
	Total	76	101	177
AI vs 2D PR	AI negative	80	7	87
	AI positive	32	58	90
	Total	112	65	177

For AI vs CBCT and 2D PR vs CBCT, CBCT was considered the reference standard. For AI vs 2D PR, conventional periapical radiography was used as the comparator. AI: Artificial intelligence; CBCT: Cone-beam computed tomography; 2D PR: Two-dimensional periapical radiography.

**Table 3:** Table 3: Diagnostic performance of AI and conventional 2D PR in detecting periapical lesions.

**Parameter**	**AI vs CBCT**	**AI vs 2D PR**	**2D PR vs CBCT**
	Value (95% CI)	Value (95% CI)	Value (95% CI)
Sensitivity	84.16% (75.55-90.67)	89.23% (79.06-95.56)	64.36% (54.21-73.64)
Specificity	93.42% (85.31-97.83)	71.43% (62.12-79.57)	100.00% (95.26-100.00)
Positive predictive value	94.44% (87.89-97.55)	64.44% (57.20-71.08)	100.00% (94.48-100.00)
Negative predictive value	81.61% (73.82-87.47)	91.95% (84.90-95.87)	67.86% (61.90-73.29)
Accuracy	88.14% (82.44-92.50)	77.97% (71.13-83.84)	79.66% (72.97-85.33)

AI: Artificial intelligence; CBCT: Cone-beam computed tomography; 2D PR: Two-dimensional periapical radiography; CI: Confidence interval. For AI vs CBCT and 2D PR vs CBCT, CBCT was used as the reference standard. For AI vs 2D PR, conventional 2D PR was used as the reference standard.

**Table 4:** Table 4: ROC analysis, agreement statistics, and inter-examiner reliability.

**Comparison**	**AUC**	**SE**	**95% CI**	**AUC ** *p * **value**	**χ²**	**χ² ** *p * **value**	**Cohen’s kappa**	**Kappa ** *p * **value**
AI vs CBCT	0.888	0.027	0.835-0.941	<0.001	2.79	0.090	0.76	<0.0001
AI vs 2D PR	0.803	0.034	0.736-0.870	<0.001	15.19	0.0001	0.56	0.001
2D PR vs CBCT	0.822	0.032	0.760-0.884	<0.001	29.88	<0.001	0.61	<0.0001
CBCT inter-examiner agreement	—	—	—	—	—	—	0.818	<0.001

Statistically significant at p<0.05. AUC: Area under the receiver operating characteristic curve; SE: Standard error; CI: Confidence interval; AI: Artificial intelligence; CBCT: Cone-beam computed tomography; 2D PR: Two-dimensional periapical radiography. Inter-examiner agreement refers to agreement between the two calibrated examiners for CBCT interpretation before consensus-based final diagnosis. The inter-examiner kappa value was 0.818, with SE = 0.043 and 95% CI: 0.734-0.902.

**Figure 1:**
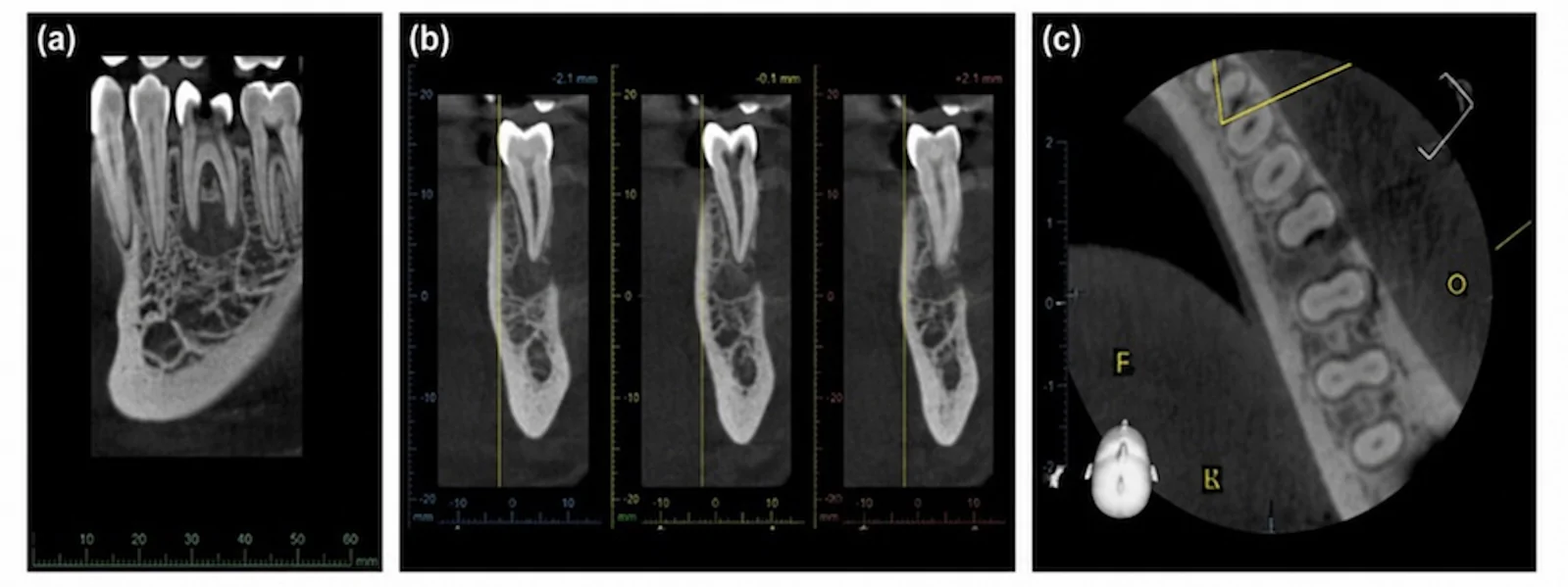
Fig. 1: Representative CBCT images demonstrating a periapical lesion in a mandibular molar: Sagittal, coronal, and axial sections.

**Figure 2:**
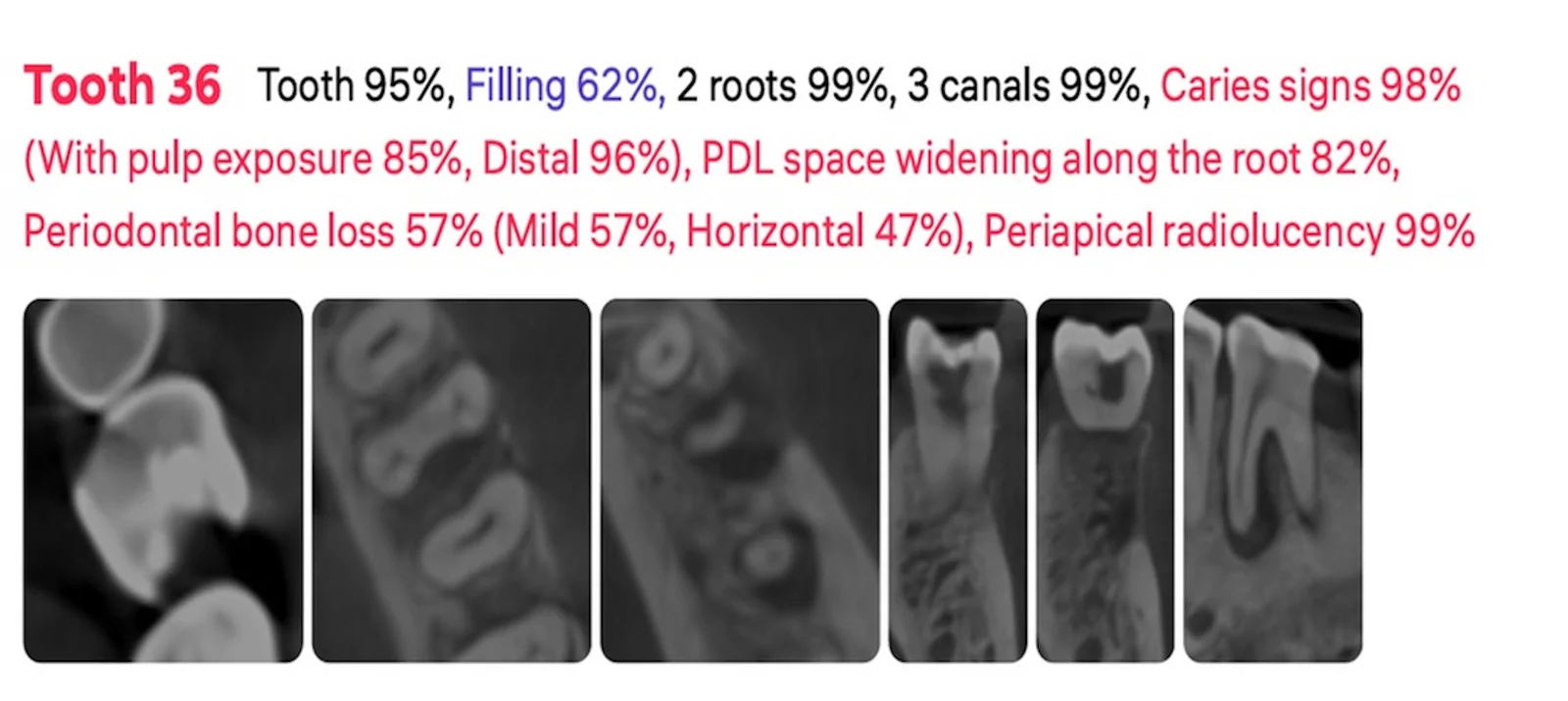
Fig. 2: AI-based radiographic analysis of tooth 36 showing automated detection of periapical radiolucency using Diagnocat software.

**Figure 3:**
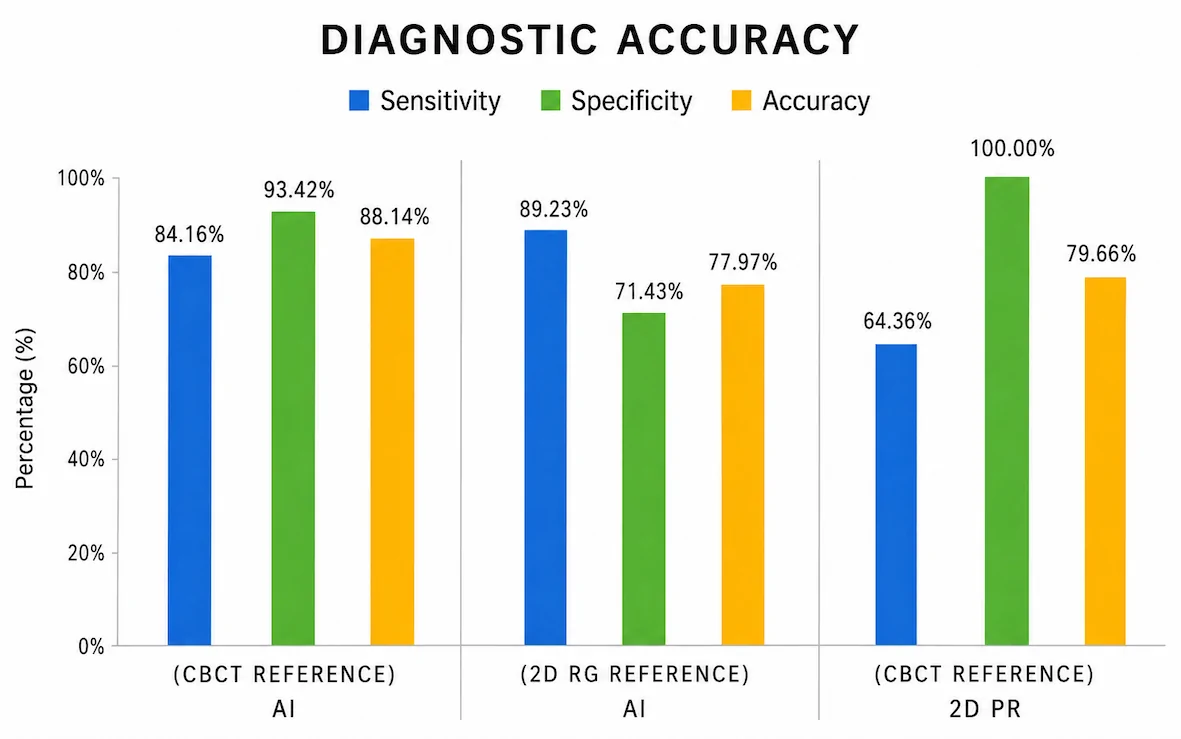
Fig. 3: Bar chart showing the sensitivity, specificity, and overall accuracy of artificial intelligence (AI) using cone-beam computed tomography (CBCT) and conventional two-dimensional radiography (2D PR) as reference standards, and the diagnostic performance of 2D PR using CBCT as the reference standard.

**Figure 4:**
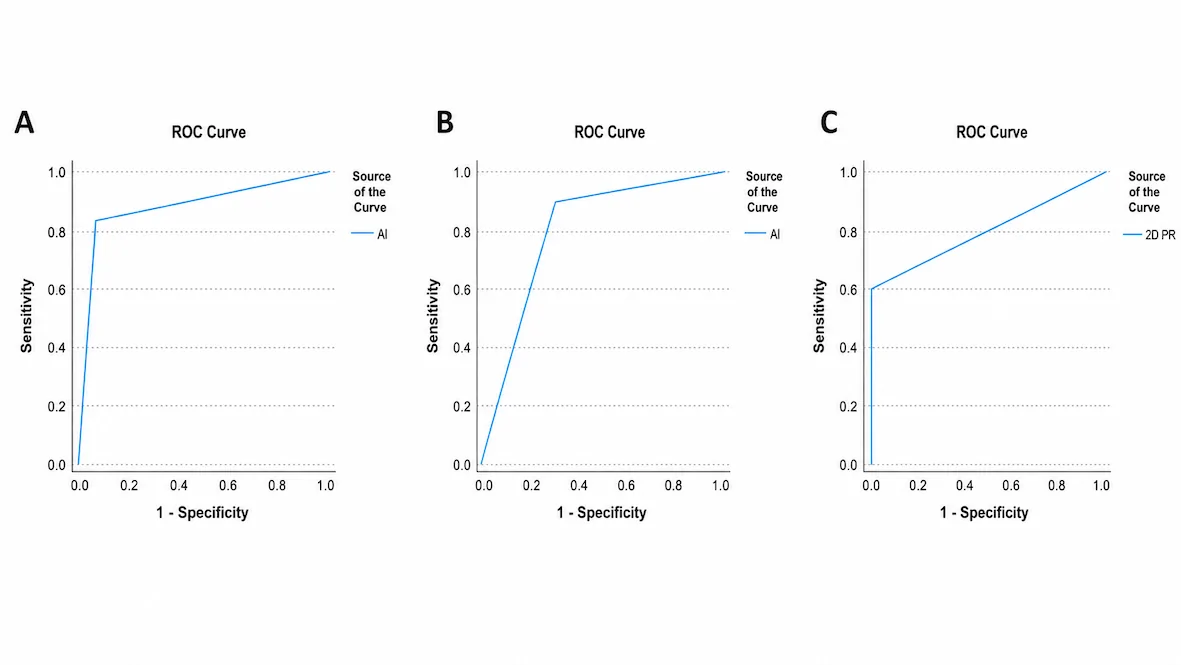
Fig. 4: Receiver operating characteristic (ROC) curve analysis of diagnostic performance for periapical lesion detection. A: ROC curve of AI using CBCT as the reference standard. B: ROC curve of AI using 2D PR as the reference standard. C: ROC curve of 2D PR using CBCT as the reference standard.

## Data Availability

Declared none.

## References

[1] Karamifar K, Tondari A, Saghiri MA (2020). Endodontic periapical lesion: An overview on the etiology, diagnosis and current treatment modalities. Eur Endod J.

[2] Arslan ZB, Demir H, Yıldız DB, Yaşar F (2020). Diagnostic accuracy of panoramic radiography and ultrasonography in detecting periapical lesions using periapical radiography as a gold standard. Dentomaxillofac Radiol.

[3] Abesi F, Golikani A (2023). Diagnostic performance of cone-beam computed tomography for apical periodontitis: A systematic review and meta-analysis. Pol J Radiol.

[4] Calazans MAA, Ferreira FABS, Alcoforado MLMG, Santos AD, Pontual ADA, Madeiro F. Automatic classification system for periapical lesions in cone-beam computed tomography. Sensors (Basel). 2022;22(17):6481. 10.3390/s22176481 PMC9459969 36080940

[5] Karobari MI, Adil AH, Basheer SN, Murugesan S, Savadamoorthi KS, Mustafa M, Abdulwahed A, Almokhatieb AA (2023). Evaluation of the diagnostic and prognostic accuracy of artificial intelligence in endodontic dentistry: A comprehensive review of literature. Comput Math Methods Med.

[6] Endres MG, Hillen F, Salloumis M, Sedaghat AR, Niehues SM, Quatela O, Hanken H, Smeets R, Beck-Broichsitter B, Rendenbach C, Lakhani K, Heiland M, Gaudin RA (2020). Development of a deep learning algorithm for periapical disease detection in dental radiographs. Diagnostics (Basel).

[7] Ourang SA, Sohrabniya F, Mohammad-Rahimi H, Dianat O, Aminoshariae A, Nagendrababu V, Dummer PMH, Duncan HF, Nosrat A (2024). Artificial intelligence in endodontics: Fundamental principles, workflow, and tasks. Int Endod J.

[8] Sadr S, Mohammad-Rahimi H, Motamedian SR, Zahedrozegar S, Motie P, Vinayahalingam S, Dianat O, Nosrat A. Deep learning for detection of periapical radiolucent lesions: A systematic review and meta-analysis of diagnostic test accuracy. J Endod. 2023;49(3):248-61.e3. 10.1016/j.joen.2022.12.007 36563779

[9] Kirnbauer B, Hadzic A, Jakse N, Bischof H, Stern D (2022). Automatic Detection of Periapical Osteolytic Lesions on Cone-beam Computed Tomography Using Deep Convolutional Neuronal Networks. J Endod.

[10] Zadrożny Ł, Regulski P, Brus-Sawczuk K, Czajkowska M, Parkanyi L, Ganz S, Mijiritsky E (2022). Artificial Intelligence Application in Assessment of Panoramic Radiographs. Diagnostics (Basel).

[11] Szabó V, Szabó BT, Orhan K, Veres DS, Manulis D, Ezhov M, Sanders A (2024). Validation of artificial intelligence application for dental caries diagnosis on intraoral bitewing and periapical radiographs. J Dent.

[12] Issa J, Jaber M, Rifai I, Mozdziak P, Kempisty B, Dyszkiewicz-Konwińska M (2023). Diagnostic test accuracy of artificial intelligence in detecting periapical periodontitis on two-dimensional radiographs: A retrospective study and literature review. Medicina (Kaunas).

[13] Hajian-Tilaki K (2014). Sample size estimation in diagnostic test studies of biomedical informatics. J Biomed Inform.

[14] Allihaibi M, Koller G, Mannocci F (2025). The detection of apical radiolucencies in periapical radiographs: A comparison between an artificial intelligence platform and expert endodontists with CBCT serving as the diagnostic benchmark. Int Endod J.

[15] Kazimierczak W, Kazimierczak N, Issa J, Wajer R, Wajer A, Kalka S, Serafin Z (2024). Endodontic treatment outcomes in cone beam computed tomography images: Assessment of the diagnostic accuracy of AI. J Clin Med.

[16] Alsaikhan LS, Algarni RA, Alzahrani MA, Gufran K, Alqahtani AM, Altammami M, Mansy I (2022). A comparative analysis of periapical status by using cone beam computed tomography and periapical radiography. Eur Rev Med Pharmacol Sci.

[17] Orhan K, Bayrakdar IS, Ezhov M, Kravtsov A, Özyürek T (2020). Evaluation of artificial intelligence for detecting periapical pathosis on cone-beam computed tomography scans. Int Endod J.

[18] Pul U, Schwendicke F (2024). Artificial intelligence for detecting periapical radiolucencies: A systematic review and meta-analysis. J Dent.

[19] Nagareddy B, Vadlamani R, Venkannagari NR, Jain S, Basheer SN, Murugesan S (2024). Comparison of the artificial intelligence versus traditional radiographic interpretation in detecting periapical periodontitis: A diagnostic accuracy study. J Pharm Bioallied Sci.

